# In Vitro Comparison of Surface Roughness, Flexural, and Microtensile Strength of Various Glass-Ionomer-Based Materials and a New Alkasite Restorative Material

**DOI:** 10.3390/polym15030650

**Published:** 2023-01-27

**Authors:** Alper Kaptan, Fatih Oznurhan, Merve Candan

**Affiliations:** 1 Department of Restorative Dentistry, University of Cumhuriyet, Sivas 58140, Türkiye; 2 Department of Pediatric Dentistry, University of Cumhuriyet, Sivas 58140, Türkiye; 3 Department of Pediatric Dentistry, University of Osmangazi, Eskişehir 26040, Türkiye

**Keywords:** Cention N, alkasite restorative, self-cured restorative, resin-modified glass ionomer cement, microtensile bond strength

## Abstract

This study aims to evaluate the physical properties of Cention N and various glass-ionomer-based materials in vitro. The groups were obtained as follows: Group 1 (LC-Cent): light-cured Cention N; Group 2 (SC-Cent): self-cured Cention N; Group 3 (COMP): composite (3M Universal Restorative 200); Group 4 (DYRA): compomer (Dyract XP); Group 5 (LINER): Glass Liner; Group 6 (FUJI): FujiII LC Capsule; and Group 7 (NOVA): Nova Glass LC. For the microtensile bond strength (μTBS) test, 21 extracted human molar teeth were used. The enamel of the teeth was removed, and flat dentin surfaces were obtained. Materials were applied up to 3 mm, and sticks were obtained from the teeth. Additionally, specimens were prepared, and their flexural strength and surface roughness (Ra) were evaluated. Herein, data were recorded using SPSS 22.0, and the flexural strength, μTBS, and Ra were statistically analyzed. According to the surface roughness tests, the highest Ra values were observed in Group 6 (FUJI) (0.33 ± 0.1), whereas the lowest Ra values were observed in Group 2 (SC-Cent) (0.17 ± 0.04) (*p* < 0.05). The flexural strengths of the materials were compared, and the highest value was obtained in Group 2 (SC-Cent) (86.32 ± 15.37), whereas the lowest value was obtained in Group 5 (LINER) (41.75 ± 10.05) (*p* < 0.05). When the μTBS of materials to teeth was evaluated, the highest μTBS was observed in Group 3 (COMP) (16.50 ± 7.73) and Group 4 (DYRA) (16.36 ± 4.64), whereas the lowest μTBS was found in Group 7 (NOVA) (9.88 ± 1.87) (*p* < 0.05). According to the μTBS results of materials-to-materials bonding, both Group 2 (SC-Cent) and Group 1 (LC-Cent) made the best bonding with Group 3 (COMP) (*p* < 0.05). It can be concluded that self-cured Cention N had the highest flexural strength and lowest surface roughness of the seven materials tested. Although the bond strength was statistically lower than conventional composites and compomers, it was similar to resin-modified glass ionomer cements. Additionally, the best material-to-material bonding was found between self-cured Cention N and conventional composites.

## 1. Introduction

Masticatory forces, oral environment, oral habits, and physical properties of dental restorations play a crucial role in obtaining long-term restorations, and therefore, a healthy tooth structure. Dental amalgam, which has excellent physical properties, has been used as a dental restoration material for many years. However, it has many disadvantages, such as poor esthetics, leakage, postoperative sensitivity, plaque accumulation, tooth coloration, and difficulty in cavity preparation [1]. To overcome these disadvantages, various dental restorative materials are being developed. For example, composite resins are widely used by dental professionals. Nevertheless, they can cause many clinical problems, such as polymerization shrinkage, marginal leakage and discoloration, excessive surface loss, side effects due to monomer release, and bacterial adhesion [2,3].

Glass ionomer cements are also frequently preferred restorative materials in dentistry. Physicochemical bonding to enamel and dentin, biocompatibility with dental tissues, fluoride ion release properties, and low thermal expansion coefficients similar to tooth structure are its positive features. However, conventional glass ionomer cements also have negative properties, such as being affected by moisture, having an opaque color, low flexural strength, and high surface roughness. To overcome these negative properties, polymerizable resin-based glass ionomer materials have been developed. Thus, the setting time of glass ionomer cements is shortened, early moisture sensitivity is reduced, and better mechanical properties are exhibited [4].

Cention N, known as alkasite, was introduced as a new resin-based material containing alkacid fillers, such as fluoride, calcium, and hydroxide ions, to neutralize acids [5,6,7]. This material, which comprises powder and liquid phases, can be polymerized both by itself and by light after mixing the powder and liquid phases. The self-curing process is based on an initiator system consisting of a copper salt, a peroxide, and a thiocarbamide. The liquid part of Cention N contains hydroperoxide, and the standard filler in the powder part of the product is coated with the other initiator components. The copper salt accelerates the curing reaction. Additionally, Cention N contains the photoinitiator Ivocerin^®^ and an acyl phosphine oxide initiator for optional light-curing, with a dental polymerization unit [8]. It is a restorative material based on urethane dimethacrylate (UDMA), which allows it to polymerize with light upon request, and it can also polymerize by itself. It is radio-opaque and contains alkaline glass fillers that can release fluoride, calcium, and hydroxide ions. Cention N is relatively more affordable and easier to use than the restorative materials available on the market [9]. Chole et al. [10] reported in their study that the flexural strength of Cention N is higher than that of light-cured composite resin and resin-modified glass ionomer. Due to the limited information available in the literature about Cention N more researches are needed on the use of this restoration material, particularly in primary teeth.

Physical properties are important factors for dental materials and also for restorative material selection in dental treatments. Since there are few studies about Cention N, this study aimed to compare the physical properties of Cention N with different glass-ionomer-based resin materials and a conventional composite. As Cention N is marketed as a posterior restorative, another aim of this study was to evaluate bonding strength when Cention N was used as a base material.

The null hypotheses tested were that (a) no difference exists between all restorative materials tested in terms of surface roughness, flexural strength, and microtensile bond strength, and that (b) no difference exists between the LC-CENT and SC-CENT bonding strength to other materials tested.

## 2. Materials and Methods

Ethical approval was obtained from the Clinical Research Ethics Committee of Sivas Cumhuriyet University (2020-01/42). Informed consent was obtained from all the patients for the collection of extracted teeth and their use in the in vitro study.

Our study consisted of 3 parts according to test methods (surface roughness test, flexural strength test, and microtensile bond strength (μTBS) test). The sample for each subgroup consisted of 10 specimens, and the power analysis revealed *p* = 0.90145 (α = 0.01, β = 0.10, 1 − β = 0.90).

Seven experimental groups were obtained in this study as follows:

Group 1 (LC-Cent): light-cured Cention N (Ivoclar Vivadent, Schaan, Liechtenstein);

Group 2 (SC-Cent): self-cured Cention N (IvoclarVivadent, Schaan, Liechtenstein);

Group 3 (COMP): 3M Universal Restorative 200 (3M ESPE, St Paul, MN 55144, USA);

Group 4 (DYRA): Dyract XP (Dentsply, Konstanz, Germany);

Group 5 (LINER): Glass Liner (Willmann & Pein GmbH, Barmstedt, Germany);

Group 6 (FUJI): Fuji II LC Capsule (GC, Tokyo, Japan);

Group7 (NOVA): Nova Glass LC. (Imicryl, Konya, Türkiye)

The technical profiles and compositions of dental restorative materials are listed in Table 1.

### 2.1. Surface Roughness Tests

Ten specimens were prepared for each group in a Teflon plastic mold (diameter and thickness of 8 and 2 mm, respectively). For each specimen, a plastic mold was placed on flat glass, and materials were applied by a single investigator (A.K.) according to the manufacturer’s instructions. The excess material was removed using a mylar matrix strip. Except for the self-cured Cention N group, all the specimens were light-cured for 20 s with a power of 1200 mW/cm^2^ using a second-generation LED device (Elipar S10 TM, 3M ESPE, St Paul, MN, USA) following the manufacturer’s instructions. The light-curing device was positioned centrally 1 mm above the specimens. The light intensity was verified using a radiometer (Demetron LC, Kerr, Brea, CA, USA). The self-cured Cention N specimens were allowed to polymerize for 24 h in a dark chamber.

After the materials were polymerized, all the specimens were tested using a profilometer (Mitutoyo, Surftest SJ-301, Kawasaki, Japan). For each specimen, three measurements at randomly [11] different locations, with a cut-off length of 25 μm and 2 mm tracing length, and the average Ra values were recorded and analyzed using the SPSS program.

Scanning electron microscopy (SEM) images were taken from randomly chosen specimens from all groups. The specimens were coated with gold (Quorum Q150R ES, Quorum Technologies, Lewes, UK) and evaluated using SEM (Tescan MIRA3 XMU, Brno, Czech Republic). The entire sample surface was scanned and photographed at a magnification of 2000× with an accelerating voltage of 15 kV.

### 2.2. Flexural Strength

Ten specimens were prepared in a stainless-steel mold (2 × 2 × 25 mm^3^) for each experimental group. Materials were applied by a single investigator (A.K.) according to the manufacturer’s instructions and light-cured with an LED device (Elipar S10 TM, 3M ESPE, St Paul, MN, USA) as in the surface roughness test. Self-cured Cention N was removed from the mold after 24 h. The specimens were tested using a universal testing machine (LF Plus, LLOYD Instruments, Ametek, Inc., Bognor Regis, UK) with a crosshead speed of 0.5 mm/min, and the data were recorded using the SPSS program.

### 2.3. Microtensile Bond Strength (μTBS)

Twenty-one freshly extracted human third molars were used. The teeth were stored in saline solution at 4 °C and used within 1 month. All the root surfaces were cleaned to remove organic debris and deposits. One-third of the coronal teeth were removed using an Isomet low-speed diamond saw (Isomet, Buehler, Lake Bluff, IL, USA). A stereomicroscope was used to check the absence of enamel and pulp tissue on the resultant substrate. After grinding the residual occlusal enamel on wet #180 grit SiC paper, flat dentin surfaces were exposed. The exposed dentin surfaces were further polished with wet #600 grit SiC paper for 60 s to standardize the smear layer. Self-etch adhesive (AdperTM Easy One, 3M ESPE, Seefeld, Germany) was applied according to the manufacturer’s instructions.

The prepared tooth samples were divided into seven groups randomly. Materials were applied by a single investigator (A.K.) according to the manufacturer’s instructions. Dental restorative materials (up to 3 mm) were applied to the teeth with the help of a stainless-steel mold, and to standardize the application pressure, a 2 mm thick and 420 g circular glass slice was placed on top of the composite applied samples [12]. Afterward, in Group 1, 3–7 samples were light-cured immediately, and Group 2 samples waited in a dark chamber for self-polymerization. Polymerized samples were embedded in acrylic blocks. Samples of μTBS tests were prepared following the ISO/TS 11405:2015 guideline. Each tooth was sectioned in the *x* and *y* directions with a slow-speed saw under water cooling, and 10 square-shaped (1 × 1 mm^2^) sticks were obtained for each group (*n* = 10). Failed samples while obtaining the specimens before the μTBS test were not included in this study. The distribution of the failed samples per each group was as follows: Group 1=5, Group 2=4, Group 3=6, Group 4=5 Group 5=5, Group 6=6, and Group 7=4. A total of 10 obtained sticks per each group and a total of 70 sticks for 7 groups were stored in distilled water for 24 h and then fixed to a microtensile device with cyanoacrylate adhesive plus an accelerator (404 Super Cyanoplast, 404 Kimya, İstanbul, Turkey). The specimens were stressed under tension until failure using a microtensile testing machine (LF Plus, LLOYD Instruments, Ametek Inc., Bognor Regis, UK) with a crosshead speed of 0.5 mm/min; μTBS values were calculated and expressed in megapascals (MPa).

Additionally, microtensile tests were conducted to see the relationship with other materials in the use of Cention N as a base material. In this step, 4 × 4 × 4 mm^3^ cubes were obtained, and all materials (from Groups 3 to 7) were restored on Group 1 and Group 2 samples with 4 × 4 × 4 mm^3^ dimensions. After obtaining 4 × 4 × 8 mm^3^ specimens, they were embedded in acrylic blocks. The specimens were sectioned perpendicularly to the bonding surface in the *x* and *y* directions with a low-speed saw under water cooling, and 10 sticks (1 × 1 × 8 mm^3^) were obtained for each group. The sticks were stressed in tension until failure using a microtensile testing machine.

### 2.4. Statistical Analysis

The data were processed using SPSS for Windows (version 22.0; SPSS Inc., Chicago, IL, USA). The mean and standard deviation of the flexural and μTBS and surface roughness was calculated for each group. Kolmogorov–Smirnov and Shapiro–Wilk tests were used to investigate the normality of data. Since the distribution of the data was normal, it was decided to use parametric tests. The physical properties of the dental restorations (flexural and microtensile bond strength and surface roughness) were analyzed using one-way ANOVA, and multiple comparisons were performed using Tukey’s post hoc test. Statistical significance was set at *p* < 0.05.

## 3. Results

**Figure 1:**
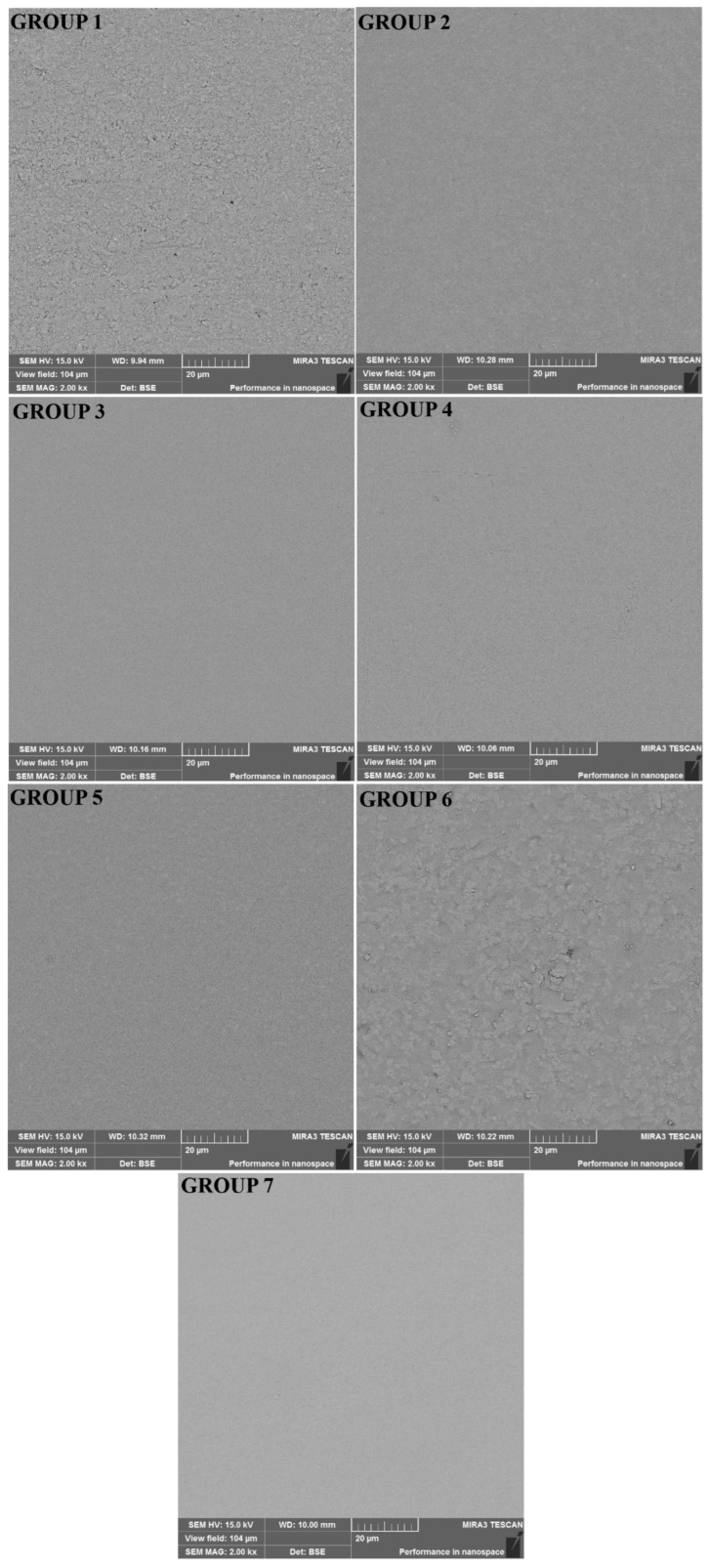
The SEM photographs obtained at 2000× magnification from the surfaces of the dental restorative materials. Group 1 (LC-Cent), Group 2 (SC-Cent), Group 3 (COMP), Group 4 (DYRA), Group 5 (LINER), Group 6 (FUJI), and Group 7 (NOVA).

When the Ra values were compared, the highest value was observed in Group 6, whereas the lowest value was observed in Group 2. A statistically significant difference was observed between the Ra values of the materials (*p* ≤ 0.05). The main Ra values and double comparisons of the materials are listed in Table 2. In addition, SEM photographs obtained at 2000× magnification from the surfaces of the materials are shown in Figure 1.

When the flexural strength values of the materials were compared, the highest value was observed in Group 2, whereas the lowest value was observed in Group 5. A statistically significant difference was observed between the flexural strength values of the materials (*p* ≤ 0.05). The main flexural strength values and double comparisons of the materials are listed in Table 2.

A statistically significant difference was found between the μTBS values of the materials to dentin bonding (*p* ≤ 0.05). When μTBS test values were compared, the highest values were observed in Group 3 and Group 4, while the lowest was found in Group 7. The mean μTBS test values of the materials and their comparisons are presented in Table 3.

When comparing the bond strength of other restorative materials to Group 1 (LC-CENT), Group 3 (COMP) bonding to LC-CENT showed the highest μTBS values, and Group 6 (FUJI) and Group 7 (NOVA) bonding to LC-CENT showed the lowest μTBS values (*p* < 0.05). No statistically significant difference was found between the μTBS values to LC-CENT material between Group 5 (LINER) and Group 4 (DYRA).

The μTBS values between the SC-CENT material and all other materials were found to be higher than those between the LC-CENT material and all the other materials. When comparing the bond strength of other restorative materials to SC-CENT, the highest μTBS values were found in Group 3 (COMP) bonding to SC-CENT, while Group 6 (FUJI) bonding to SC-CENT showed the lowest μTBS values (*p* < 0.05). There was no statistically significant difference between Group 4 (DYRA), Group 5 (LINER), Group 6 (FUJI), and Group 7 (NOVA) to SC-CENT bonding (*p* > 0.05). The main μTBS values between the dental restorative materials and self and light-cured Cention N materials, and their double comparisons are shown in Table 4.

## 4. Discussion

Both null hypotheses were rejected. The surface roughness, flexural strength, and μTBS values of the tested materials were found to be different. In addition, the bond strength of LC-CENT and SC-CENT to other tested restorative materials was found to be different.

### 4.1. Surface Roughness

Surface roughness is an important feature affecting biofilm formation in dental materials. Bacterial adhesion occurs over time with the formation of biofilms on dental surfaces. The number of microorganisms adhering to the restorative material depends on different factors, such as surface roughness, the hydrophobicity of the material surface, matrix type, electrostatic forces, material composition, filler size, and the configuration of fillers [13]. The surface roughness of dental restorative materials can be affected by internal factors, such as differences in the size, shape, volume, and distribution of inorganic fillers, as well as external factors, such as medications and liquids to which the materials are exposed [14,15,16]. As the filler size of the materials increases, the vertical surface roughness increases [17]. In addition, the inadequate polymerization of the materials may affect the average surface roughness values of dental restorative materials. In a previous study, the surface roughness value of the self-cured form of the glass ionomer material was lower than that of the light-cured form [18,19]. However, there was no statistically significant difference between the groups polymerized using different methods. Similarly, in the present study, the self-cured form of Cention N was smoother than the light-cured form [13].

Various methods can be used to polish the surfaces of dental restorative materials. The smoothest surface can be obtained using mylar strip bands [20,21]; therefore, in the present study, mylar strips were used to obtain a flat surface [22]. Setty et al. [23] compared composites’ and Cention’s surface roughnesses and found that the composites showed better results than light-cured cement. According to the results of this study, self-curing Cention and composites showed lower values, but light-cured Cention showed higher values than those reported by Setty et al. [23] In addition, acceptable Ra values should be less than 0.2 µm [24], and both self-cured Cention and composite showed this result. Further, self-cured Cention showed lower Ra values than light-cured Cention, probably because self-cured Centions are being cured slowly and for a long time period.

### 4.2. Flexural Strength

Flexural strength tests are important because they measure occlusal forces in the oral cavity. In this study, the flexural strengths of composite resin and self-cured Cention were 83.78 and 86.32 MPa, respectively. According to the International Organization for Standardization (ISO) 4049 standards, these are acceptable values [3,7,10,24]

Mishra et al. [5] tested the flexural strength of a composite, Cention N, GIC, and amalgam, and found that the best results were obtained for the composite. Kiran et al. [25] tested the mechanical properties of Cention N and type IX GIC, and Cention N showed higher flexural strength than type IX GIC. They suggested that this result was due to the filler content and monomers used in the materials. Sadananda et al. [7] compared the flexural strengths of Cention N, Fuji IX, Ketac-Molar, and Zirconomer and found that Cention N showed the highest flexural strength, whereas Fuji IX showed the lowest flexural strength. Panpisut et al. [6] tested two resin-modified GICs, Cention N, and a composite, and found that the composite displayed the highest flexural strength in the composite group, followed by that of Cention N, Fuji II, and Riva LC. The results were similar to those of the present study, and Cention N displayed higher results than Fuji II and other GICs. Chole et al. [10] tested the flexural strength of Cention N, a bulk-fill composite, nanocomposite, and resin-modified GIC. In their study, Cention N showed the highest flexural strength, whereas resin-modified GIC showed the lowest flexural strength, which is in accordance with the results of the proposed study. The authors attributed this highest flexural strength to the material’s content and UDMA, and we agree with these authors [5,6,7,10,25]. Light-cured Cention N showed statistically lower results than self-cured Cention N, probably due to the light-cured Cention N’s fast curing with LEDs.

### 4.3. Microtensile Bond Strength

μTBS has been used in several studies and is one of the most standardized and versatile bond strength tests. In this study, the materials were tested in two parts. One is to test bonding materials to teeth, and the other is bonding materials to another restorative material to test if Cention N could be used as a base material.

In this study, the composite (16.5 MPa) and compomer (16.36 MPa) materials showed higher μTBS values than the self-cured Cention N (12.5 MPa) and light-cured (13.25 MPa) Cention N; however, Cention N showed higher values than the other GIC groups. Yao et al. [26] tested the μTBS of Cention N, a bulk-fill composite, and Fuji II LC, and found the highest scores with Cention N, and the results were similar to those of this study. Both Cention N groups showed higher results than Fuji II LC. Naz et al. [27] tested the shear bond strength of composite and Cention N, and the highest results were shown by Cention N. Similarly, Eligeti et al. [28,29] reported that Cention N restorative material showed better bonding potential to dentin compared to the bonding potentials of resin-modified GIC, Zirconomer-enhanced, and Ketac-Molar. In microtensile or shear bond strength tests, many factors affect the results, such as tooth age, depth of dentine, dentine tubules, moisture, materials, and test conditions. In this study, Cention N showed lower μTBS values than the composite and compomer when compared to those obtained in other studies, and the possible explanation for this could be the conditions listed above. Cention N showed higher μTBS values than Group 5, Group 6, and Group 7, suggesting that Cention N has a high polymer network density and good bonding ability to dentin. This may also be explained by the fact that alkasites do not contain Bis-GMA, HEMA, or TEGDMA. UDMA was the main component of the monomer sequence. A combination of UDMA, DCP, aromatic and aliphatic UDMA, and PEG–400 DMA provides enhanced mechanical properties and good long-term stability during polymerization. PEG–400 DMA is a monomer liquid that increases the fluidity of the material, and its hydrophilic character supports the ability of the material to wet the substrate (enamel and dentin) and adapt to the smear layer [30].

To the best of our knowledge, this is the first study to use Cention N as a base material. Self-cured Cention N showed higher results than light-cured Cention N when bonded with other materials, and it was significantly different when bonded with Nova and Fuji II LC. This can primarily be attributed to the fact that while Cention N is self-curing, it provides stronger bond strengths.

Butera et al. [31] showed calcium and phosphorus ions’ deposition on the surfaces of bulk-filled polymeric composite resins in the oral environment after one month of daily oral hygiene application with a toothpaste containing microRepair^®^ (Zn-carbonate hydroxyapatite). Since Cention N is an ion-releasing restorative material, it may be a future goal to investigate whether the use of biomimetic hydroxyapatite will be effective in increasing ion deposition in this restorative material and reducing the incidence of secondary caries.

This study has some limitations. Firstly, only one material was used in the self-cure mode, which may have the potential effect to decrease the generalizability of findings. Further studies should examine the effect of different types of self-cured restorative materials. Another limitation was that this study was carried out under in vitro conditions. However, in the clinical use of this restorative material, the presence of saliva in the oral environment, visibility problems, transport of the material into the cavity, variable mixing rates, difficulties in the proximal cavities, and the need to complete the restoration in a short time may reduce the performance of this restorative material in oral conditions. In this context, there is a need for long-term clinical studies to evaluate the clinical performance of this material. Within the limitations of this study, Cention N showed superior results in all three tests when compared to conventional and resin-modified glass ionomer cements in in vitro conditions. Additionally, self-cured Cention N showed enhanced results when compared to those shown by light-cured Cention N.

## 5. Conclusions

Although self-cured Cention N had promising results with the highest flexural strength and lowest surface roughness of the seven materials tested, bond strength to dentin values were statistically lower than conventional composites and compomers, displaying similar strengths to resin-modified glass ionomer cements. These findings make the use of this material as a permanent restorative material questionable. However, considering that the best material–material bond in the present study is between self-cured Cention N and conventional composites, and when the ion-release (F^−^, OH^−^ and Ca^2+^) feature to induce the incidence of secondary caries of this material is taken into account, it can be considered that Cention N may be more suitable for use as a base material under composite materials. Further in vivo and in vitro studies are necessary to validate these findings. Because Cention N is a newly developed material, the present study is one of the few studies that investigate the mechanical properties of this material.

## Figures and Tables

**Table 1:** The technical profiles, codes, and compositions of the dental restorative materials.

Materials	Code	Composition	Manufacturer	LOT Number
Universal Restorative 200	COMP	Bis-GMA, Bis-EMA, UDMA, silica/zirconia, Filler 60% (volume)	3M ESPE, St Paul, MN 55144, USA	NA06972
Dyract XP	DYRA	UDMA, TCB resin, TEGDMA, trimethacrylate resin. 73 wt% Strontiumalumino-sodium-fluoro-phosphor-silicate	Dentsply, Konstanz, Germany	2003000720
Nova Glass LC	NOVA	*Powder:* Floro Alumino Silicate Glass, Pigments *Liquid:* Composite m resins 25–27% (Hema, dimethacrylates), Catalists, Stabilisators	Imicryl, Konya, Türkiye	20031
Fuji II LC Capsule	FUJI	2-hydroxyethyl methacrylate, Polyacrylic acid, and water. 58 wt% Fluoro-aluminumsilicate	GC, Tokyo, Japan	1908281
Glass Liner	LINER	Glasionomerpulver, 1,6-Hexandioldimethacrylate, Bisphenol-A-bis (hydroxypropylmethacrylat), Isomere, 4-tert.-Butyl-N,N-dimethylaniline, Campherchinon	Willmann & Pein GmbH, Barmstedt, Germany	187576
Cention N	CENT	*Powder:* Barium aluminum silicate glass, ytterbium trifluoride, isofiller, calcium barium aluminum fluorosilicate glass, and calcium fluorosilicate glass *Liquid:* Urethane dimethacrylate, tricyclodecane dimethanol dimethacrylate, tetramethyl-xylylen diurethane dimethacrylate, polyethylene glycol 400 dimethacrylate, Ivocerin, and hydroxyperoxide	Ivoclar Vivadent, Schaan, Liechtenstein	Z0054T
AdperTM Easy One		2 HEMA, Bis-GMA, Methacrylated 85010 phosphoric esters, 1,6 hexaneddiol dimethacrylate, Methacrylate functionalized polyalkenoic acid (vitrebond copolymer), dispersed bonbed silica fillers 7 nm, ethanol, water, camphorquinone, stabilizers. pH = 2.4	3M ESPE, Seefeld, Germany	6744628

**Table 2:** Surface roughness and flexural strength values of the dental restorative materials.

Materials	Mean ± Standard Deviation (µm)	Mean ± Standard Deviation (MPa)
Group 1 (LC-CENT)	0.27 ± 0.04 ^a^	58.17 ± 8.38 ^a^
Group 2 (SC-CENT)	0.17 ± 0.04 ^b^	86.32 ± 15.37 ^b^
Group 3 (COMP)	0.18 ± 0.06 ^b^	83.78 ± 16.65 ^b^
Group 4 (DYRA)	0.22 ± 0.1 ^b^	61.21 ± 9.82 ^a^
Group 5 (LINER)	0.18 ± 0.07 ^b^	41.75 ± 10.05 ^c^
Group 6 (FUJI)	0.33 ± 0.1 ^c^	48.17 ± 6.24 ^d^
Group 7 (NOVA)	0.26 ± 0.07 ^a^	57.83 ± 22.98 ^a,d^

In each column, groups with the different lowercase superscripts are significantly different (*p* < 0.05).

**Table 3:** The microtensile bond strength (μTBS) values for each dental restorative material with teeth.

Materials	Mean ± Standard Deviation (MPa)	
Group 1 (LC-CENT)	13.25 ± 4.4 ^B^	F = 2.873 *p* = 0.015 *
Group 2 (SC-CENT)	12.50 ± 5.05 ^B^	
Group 3 (COMP)	16.50 ± 7.73 ^A^	
Group 4 (DYRA)	16.36 ± 4.64 ^A^	
Group 5 (LINER)	11.98 ± 5.01 ^B^	
Group 6 (FUJI)	11.17 ± 3.0 ^B^	
Group 7 (NOVA)	9.88 ± 1.87 ^C^	

The different uppercase letters represent the difference in the columns. * *p* < 0.05 was accepted as the significance level.

**Table 4:** Microtensile bond strength (μTBS) values of dental restorative materials bonding to light and self-cured Cention N specimens.

Materials	Group 1 (LC-CENT)	Group 2 (SC-CENT)	
	Mean ± Standard Deviation (MPa)		*p* Values
Group 3 (COMP)	20.00 ± 3.09 ^a^	23.69 ± 6.68 ^a^	*p* = 0.131
Group 4 (DYRA)	17.23 ± 2.53 ^a,b^	20.09 ± 5.59 ^a,b^	*p* = 0.156
Group 5 (LINER)	15.24 ± 2.20 ^b,c^	17.39 ± 2.94 ^a,b^	*p* = 0.081
Group 6 (FUJI)	11.52 ± 2.65 ^c^	16.26 ± 5.09 ^b^	*p* = 0.018 *
Group 7 (NOVA)	12.79 ± 4.99 ^c^	21.72 ± 3.95 ^a,b^	*p* = 0.001 *

* In each column, groups with the different lowercase superscripts are significantly different (*p* < 0.05).

## Data Availability

The data presented in this study are available within this article.
